# Using Weighted Sparse Representation Model Combined with Discrete Cosine Transformation to Predict Protein-Protein Interactions from Protein Sequence

**DOI:** 10.1155/2015/902198

**Published:** 2015-10-28

**Authors:** Yu-An Huang, Zhu-Hong You, Xin Gao, Leon Wong, Lirong Wang

**Affiliations:** ^1^College of Computer Science and Software Engineering, Shenzhen University, Shenzhen, Guangdong 518060, China; ^2^School of Computer Science and Technology, China University of Mining and Technology, Xuzhou, Jiangsu 221116, China; ^3^Department of Medical Imaging, Suzhou Institute of Biomedical Engineering and Technology, Suzhou, Jiangsu 215163, China; ^4^School of Electronic and Information Engineering, Soochow University, Suzhou, Jiangsu 215123, China

## Abstract

Increasing demand for the knowledge about protein-protein interactions (PPIs) is promoting the development of methods for predicting protein interaction network. Although high-throughput technologies have generated considerable PPIs data for various organisms, it has inevitable drawbacks such as high cost, time consumption, and inherently high false positive rate. For this reason, computational methods are drawing more and more attention for predicting PPIs. In this study, we report a computational method for predicting PPIs using the information of protein sequences. The main improvements come from adopting a novel protein sequence representation by using discrete cosine transform (DCT) on substitution matrix representation (SMR) and from using weighted sparse representation based classifier (WSRC). When performing on the PPIs dataset of *Yeast*, *Human*, and *H. pylori*, we got excellent results with average accuracies as high as 96.28%, 96.30%, and 86.74%, respectively, significantly better than previous methods. Promising results obtained have proven that the proposed method is feasible, robust, and powerful. To further evaluate the proposed method, we compared it with the state-of-the-art support vector machine (SVM) classifier. Extensive experiments were also performed in which we used *Yeast* PPIs samples as training set to predict PPIs of other five species datasets.

## 1. Introduction

Proteins are the molecules which participate in virtually every aspect of cellular function within an organism and responsible for the majority of the activities of living cells. Usually, proteins rarely carry out their functions alone. For example, structural proteins need to work in pairs to shape organelles and the whole cell, and the same is true for ribosome, RNA polymerases, and multisubunit channels in membranes. Detecting protein-protein interactions (PPIs) can provide a great insight into molecular mechanisms of biological processes and promote the practical medical applications based on those mechanisms. Much effort has been devoted to identifying protein interaction using high-throughput technologies such as yeast two-hybrid (Y2H) screens [1, 2], tandem affinity purification (TAP) [3], and mass spectrometric protein complex identification (MS-PCI) [4]. However, these experimental methods are still time-consuming and expensive. In addition, they yield many false positives and can only identify a small fraction of the whole protein interaction network. For this reason, the issue of predicting unknown PPIs is now considered hard to be solved only by using experimental methods.

For the sake of utilizing the available PPIs data experimentally obtained, it is of much significance to develop computational methods for predicting protein-protein interactions. A number of experiments which depict PPI networks of living organism have been finished and a number of datasets such as MINT [5], BIND [6], and DIP [7] have been built to store proteins interaction data. However, the quantities of these different kinds of available protein data such as protein sequences, secondary structures, and tertiary structures are in different levels. Protein sequence data hold a great advantage in quantitative term. With the exponential growth of newly discovered protein sequence data, it is increasingly important to develop computational methods using the information of amino acid sequences. Sequence-based computational approaches usually contain two steps: feature extraction and sample classification [8–13].

Feature extraction from protein sequence aims to mine the most representative attributes from the samples and to normalize different-length protein sequences to vectors of the same size. Efficient feature descriptors are capable of improving the performance of classification model [10, 14]. Until now, a number of feature extraction methods based on protein sequences have been proposed. Most of these methods are based on Chou's pseudoamino acid composition (PseAAC) [15, 16]. PseAAC expends the simple amino acid composition (AAC) by considering and retaining the information of sequence order. Different kinds of feature descriptors based on PseAAC prove to be powerful and become popular in protein feature extraction. However, some other feature extraction methods have put forward new ways which are based on kernels. Jaakkola et al. [17] have first proposed Fisher kernel for homology detection. Equally, mismatch string kernel proposed by Leslie et al. [18–20] measures sequence similarity counting the shared occurrences of subsequences in a lower computational cost. Unlike PseAAC-based feature extraction methods which extract feature directly from protein sequences, kernel-based methods remain some kinds of prior information and therefore extract more comprehensive feature descriptors.

In this work, we employ a novel kernel-based feature extraction method using the substitution matrix representation (SMR). In the process of evolution, the protein sequences gradually alter with the action of DNA mutations from one generation to the next. Thus, in the process of extracting protein sequence features, it is reasonable to consider the influence of the rate at which one character in a protein sequence changes to others over time. We adopt SMR based on BLOSUM62, which is the default matrix for protein BLAST and is considered to be powerful for detecting most weak protein similarities.

In the second step, we apply weighted sparse representation based classifier (WSRC), a variant of traditional SRC, to classify the interacting and noninteracting protein pairs based on their feature representation. Recently, sparse representation which is originated from signal processing area comes to be a new hot technique. This technique addresses pattern classification problems in a novel way and proves sufficiently robust against illumination variations, occlusions, and random noise. In addition, unlike the traditional sample classifiers such as support vector machine [21, 22] and neural network [23] which need much effort to adjust the best parameters, it needs little manual intervention to use SRC in sample classification. WSRC, which integrates both sparsity and locality structure data, can further improve the classification performance of SRC. For this reason, we use weighted sparse representation based classifier to build a computational classification system for predicting protein interaction.

In this paper, we propose a computational method for predicting PPIs from amino acid sequences combining substitution matrix representation and weighted sparse representation based classifier. More specifically, we first adopt substitution matrix representation based on BLOSUM62 to represent proteins as SMR matrixes. Secondly, we utilize discrete cosine transform to extract a 400-dimensional vector from each protein SMR matrix. As a result, each protein pair is represented by an 800-dimensional feature vector. Finally, WSRC is employed as the machine learning classifier to deal with the classification. The proposed method was evaluated by using three different PPIs datasets:* Yeast*,* Human*, and* H. pylori*. To further evaluate the performance of the proposed method, we compare it with the state-of-the-art support vector machine classifier. Extensive cross-species experiments were also performed on five independent PPIs datasets. In these experiments, we used experimentally identified interactions in one organism to predict the interactions in other five organisms assuming that homolog proteins preserve their ability to interact. The experimental results show that the proposed method performs significantly well in distinguishing interacting and noninteracting protein pairs. Achieved results demonstrate that the proposed approach outperforms all other previous methods on a couple of PPI datasets and can be a useful supplementary tool to traditional experimental method.

## 2. Materials and Methodology

### 2.1. Godden Standard Datasets

We verify the proposed method on a high-confidence* Saccharomyces cerevisiae* PPIs dataset which is gathered from publicly available database of interacting proteins (DIP). The protein pairs with less than 50 residues are removed because they might just be fragments. The protein pairs with too much sequence identity are generally considered to be homologous; thus the pairs which have ≥40% sequence identity are also deleted in order to eliminate the bias to these homologous sequence pairs. By doing this, we got the remaining 5594 protein pairs which construct the positive dataset. For constructing the negative dataset, we selected 5594 additional protein pairs of different subcellular localizations to build the negative dataset. Consequently, the whole dataset is made up of 11188 protein pairs of which half are from the positive samples and half are from the negative samples.

In order to demonstrate the generality of the proposed method, we also verify our approach on two other types of PPIs datasets. We collected the first dataset from the* Human* Protein References Database (HPRD). We removed those protein pairs which have ≥25% sequence identity. Finally, to comprise the golden standard positive dataset, we used the remaining 3899 protein-protein pairs of experimentally verified PPIs from 2502 different human proteins. For golden standard negative dataset, we followed the previous work [24] assuming that the proteins in different subcellular compartments do not interact with each other and finally obtained 4262 protein pairs from 661 different* human* proteins as the negative dataset. As a result, the* Human* dataset is constructed by 8161 protein pairs. The second PPI dataset is constructed by 2916* Helicobacter pylori* protein pairs (1458 interacting pair and 1458 noninteracting pairs) as described by Martin et al.

### 2.2. Substitution Matrix Representation

Substitution matrix representation is a variant of representation method proposed by [36]. In this novel matrix representation for proteins, a *N* × 20 matrix would be generated to represent a given *N*-length protein sequence based on a substitution matrix. In our work, we applied BLOSUM62 matrix, a popular substitution matrix used for sequence alignment of proteins, to this transformation. SMR can be depicted as follows:

(1) $$\begin{array}{c} SMR\left(\;i,j\;\right)=B\left(\;P\left(\;i\;\right),j\;\right)\;i=1\cdots N,\;\;j=1\cdots 20, \end{array}$$

where *B* denotes the BLOSUM62 which is a 20 × 20 substitution matrix and *B*(*i*, *j*) represents the probability rate of amino acid *i* mutating to amino acid *j* in the evolution process; *P* = (*p*1, *p*2 ⋯ *pN*) is the given protein sequence constructed by *N* amino acids.

### 2.3. Discrete Cosine Transform

Discrete cosine transform (DCT) first proposed by Ahmed et al. [37] is a popular linear separable transformation in the lossy signal and image compression processing for its powerful energy compaction property. In DCT algorithm, an input signal would be converted into elementary frequency components. In addition, small high-frequency components would be discarded, which can approach high compaction efficiency. Discrete cosine transform can be defined as follows:

(2) DCTi,j=kikj∑m=0M−1 ∑n=0N−1Sigm,ncos⁡π2m+1i2M·cos⁡π2n+1i2No≤i≤M,  0≤j≤N,

where

(3) ki=1M,i=0,2M,1≤i≤M−1,kj=1N,i=0,2N,1≤i≤N−1.

Sig ∈ *R* ^*N*×*M*^ is the input signal matrix and here denotes the *N* × 20 SMR matrix. In our work, the final DCT feature descriptor which represents a protein sequence is obtained by choosing the first 400 coefficients.

### 2.4. Weighted Sparse Representation Based Classification (WSRC)

With the advancement in mathematical studies on linear representation methods (LRBM) and compressed sensing (CS) theory, sparse representation has recently earned considerable attention in field of signal processing, computer vision, and pattern recognition. The sparse representation based classification (SRC) [38, 39] assumes that it is sufficient to represent a given test sample by using samples from the sample subject. Based on this viewpoint, SRC computes a sparse representation matrix in a specific optimizing strategy aiming to build a linear combination of training set to represent the given test sample. Employing the sparse representation matrix, reconstruction residuals of each class would be calculated and the test sample will be finally assigned to the class which has the minimum reconstruction residual.

Given a training sample matrix *X* ∈ *R* ^*m*×*n*^ which is made up of *nm*-dimensional training samples, assume that sufficient training samples belonging to the *k*th class. Samples of *k*th class can make up a submatrix *X*_*k*_ = [*l*_*k*1_, *l*_*k*2_ ⋯ *l*_*kn*_*k*__], where *l*_*i*_ denotes the label of *i*th sample and *n*_*k*_ is the number of samples belonging to *k*th class. Thus, the sample matrix *X* can be further rewritten as *X* = [*X*_1_ *X*_2_ ⋯ *X*_*K*_], where *K* is the class number of the whole samples. Given a test sample *y* ∈ *R* ^*m*^, SRC represents it with the linear combination of training samples of *k*th class:

(4) $$\begin{array}{c} y=\alpha_{k,1}l_{k,1}+\alpha_{k,2}l_{k,2}+\cdots +\alpha_{k,n_{k}}l_{k,n_{k}}, \end{array}$$

which can be further symbolized with the consideration of the whole training set representation as follows:

(5) $$\begin{array}{c} y=X\alpha_{0}, \end{array}$$

where *α*_0_ = [0,…,0, *α*_*k*,1_, *α*_*k*,2_,…,*α*_*k*,*n*_*k*__, 0,…,0]^*T*^. For the reason that the nonzero entries in *α*_0_ are only associated with the *k*th class, when the class number of samples is large, *α*_0_ would come to be sparse. The key of SRC algorithm is searching the *α* vector which can not only satisfy (5) but also minimize the *ℓ*_0_-norm of itself:

(6) α^0=arg min⁡α0subject  to y=Xα.

However, problem (6) is NP-hard and hard to be solved precisely. According to the theory of compressive sensing, when *α* is sparse enough, it is feasible to solve the related convex *ℓ*_1_-minimization problem to avoid solving the solution of *ℓ*_0_-minimization problem directly:

(7) $$\begin{array}{c} {\hat{\alpha }}_{1}=\arg\;\min{\left\|\;\alpha \;\right\|}_{1}\\ \text{subject to}\;y=X\alpha . \end{array}$$

Dealing with occlusion, (7) needs to be extended to the stable *ℓ*_1_-minimization problem:

(8) $$\begin{array}{c} {\hat{\alpha }}_{1}=\arg\;\min{\left\|\;\alpha \;\right\|}_{1}\\ \text{subject to}\;\left\|\;y-X\alpha \;\right\|\leq \varepsilon , \end{array}$$

where *ε* > 0 denotes to the tolerance of reconstruction error. Given the solution from (8), the SRC algorithm assigns the label of test sample *y* to class *c* based on the following rule:

(9) $$\begin{array}{c} \underset{c}{\min}\;r_{c}\left(\;y\;\right)=\left\|\;y-X{\hat{\alpha }}_{1}^{c}\;\right\|,\;c=1\cdots K. \end{array}$$

Then, traditional SRC represents a test sample as a sparse combination of training sample and assigns it to the class which minimizes the residual between itself and $X{\hat{\alpha }}_{1}^{c}$.

Nearest Neighbor (NN) is another distinct classifier which classifies the test sample by only using its Nearest Neighbor in training data. It utilizes the locality structure of data but easily suffers from noise. Locality measures the similarity between the query and training samples and comes to be a key issue in the fields of clustering, dimension reduction, density estimation, anomaly detection, and image classification. Researches [15, 40, 41] show that, in some case, locality is more essential than sparsity. Although SRC uses the linearity structure of data and overcomes the drawback of NN, the original sparse coding fails to guarantee being local which could cause instability. For this reason, it has sufficient reasons to integrate the locality structure of data into sparse representation. Lu et al. [42] have recently proposed a variant of traditional sparse representation based classifier called weighted sparse representation based classifier (WSRC). This variant classifier possesses the advantages of both the traditional sparse representation based classifier and the Nearest Neighbor classifier. Appropriate kernel methods map samples into a high-dimensional feature space and usually lead to a better performance in classification process. For this reason, WSRC first utilizes distance based on Gaussian kernel to measure the similarity between the samples. Gaussian-based distance can be described as follows:

(10) $$\begin{array}{c} d_{G}\left(\;x,y\;\right)=e^{-{\left\|x-y\right\|}^{2}/2\sigma^{2}}, \end{array}$$

where *x*, *y* ∈ *R* ^*d*^ denote two samples and *σ* is the Gaussian kernel width. These Gaussian distance values are then used as the weights of each sample in training sets and adjust training sample matrix *X* into a new matrix *X*′ [43, 44]. In this way, weight sparse representation based classifier is capable of retaining the locality structure of data. WSRC turn to solve the following problem:
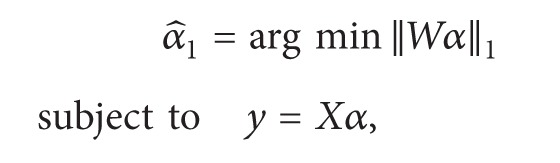
(11)

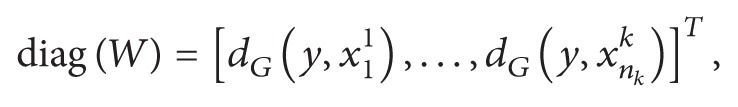
(12)where *W* is a block-diagonal matrix of locality adaptor and *n*_*k*_ is the sample number of training set in class *k*. Dealing with occlusion, WSRC finally solves the following stable *ℓ*_1_-minimization problem:

(13) $$\begin{array}{c} {\hat{\alpha }}_{1}=\arg\;\min{\left\|\;W\alpha \;\right\|}_{1}\\ \text{subject to}\;\left\|\;y-X\alpha \;\right\|\leq \varepsilon , \end{array}$$

where *ε* > 0 is the tolerance value.

Given all these, the WSRC algorithm can be summarized as follows.

**Algorithm 1 .**

Weighted Sparse Representation Based Classifier (WSRC)

(1) Input: training samples matrix *X* ∈ *R* ^*m*×*n*^ and any test sample *y* ∈ *R* ^*d*^.
(2) Normalize the columns of *X* to have unit *ℓ*_2_-norm.
(3) Calculate the Gaussian distances between *y* and each sample in *X* and make up matrix *W*. Use matrix *W* to adjust the training samples matrix *X* to *X*′.
(4) Solve the stable *ℓ*_1_-minimization problem defined in (12).
(5) Compute each residual of *K* classes: $r_{c}(y)=\left\|y-X{\hat{\alpha }}_{1}^{c}\right\|$ (*c* = 1,2,…, *K*).
(6) Output: the prediction label of *y* as identity(*y*) = arg min_*c*_⁡(*r*_*c*_(*y*)).

## 3. Results and Discussion

### 3.1. Evaluation Measures

To evaluate the performance of the proposed method, we use the following criteria: the overall prediction accuracy (Accu.), sensitivity (Sens.), precision (Prec.), and Matthews's correlation coefficient (MCC) were calculated. They are defined as follows:

(14) Accuracy=TP+TNTP+FP+TN+FNSensitivity=TPTP+FNPE=TPTP+FPMCC=TP×TN−FP×FNTP+FN×TN+FP×TP+FP×TN+FN,

where true positive (TP) denotes the number of true samples which are predicted correctly; false negative (FN) is the number of true samples predicted to be noninteracting pairs incorrectly; false positive (FP) is the number of true noninteracting pairs predicted to be PPIs falsely; and true negative (TN) is the number of true noninteracting pairs predicted correctly. Furthermore, the receiver operating characteristic (ROC) curves are also computed for evaluating the performance of proposed method. Summarizing ROC curve in a numerical way, the area under an ROC curve (AUC) is computed.

### 3.2. Assessment of Prediction Ability

For the sake of fairness, the corresponding parameters of weighted sparse representation based classifier were set the same when explored in three different datasets—*Yeast*,* Human*, and* H. pylori*. Here, *σ* = 1.5 and *ε* = 0.00005. In addition, 5-fold cross-validation was employed in our experiments in order to avoid the overfitting of the prediction model and test the stability of the proposed model [45]. Specifically, the whole dataset was divided into five parts where four parts were used for training and one part was used for testing. In this way, we obtained 5 models from the original dataset and each one of them was experimented solely. The prediction results of SRC prediction models with substitution matrix representation based description of protein sequence on three datasets are shown in Tables 1–3.

**Figure 1:**
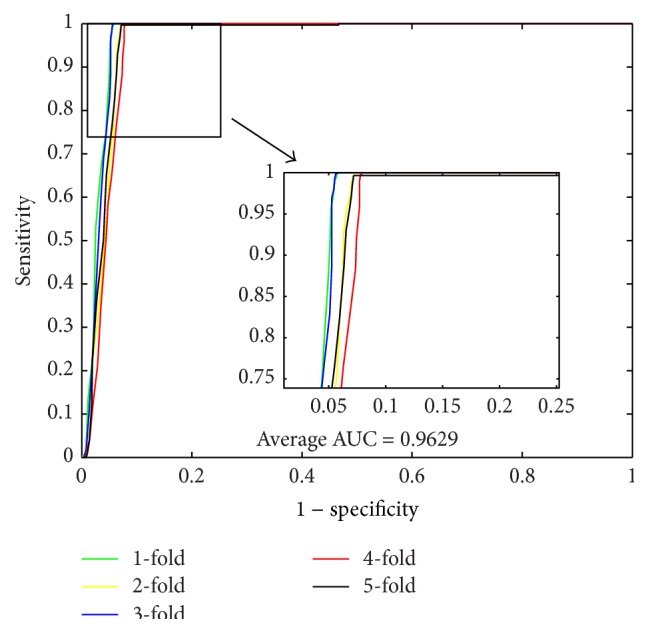
ROC curves performed by proposed method on* Yeast* PPIs dataset.

When using the proposed method to predict PPIs of* Yeast* dataset, we obtained the results of average accuracy, precision, sensitivity, and MCC of 96.28%, 99.92%, 92.64%, and 92.82%. The standard deviations of these criteria values are 0.52%, 0.18%, 1.00%, and 0.97%, respectively. When predicting PPIs of* Human* dataset, the proposed method yielded good results of average accuracy, precision, sensitivity, and MCC of 96.30%, 99.59%, 92.63%, and 92.82% and the standard deviations are 0.10%, 0.29%, 0.44%, and 0.19%, respectively. When predicting PPIs of* H. pylori* dataset, the averages of accuracy, precision, sensitivity, and MCC come to be 86.74%, 87.01%, 86.43%, and 76.99% and the standard deviations are 1.52%, 2.23%, 0.86%, and 2.23%, respectively. The ROC curves performed on these three datasets are shown in Figures 1, 3, and 5. In these figures, *x*-ray depicts false positive rate (FPR) while *y*-ray depicts true positive rate (TPR). To further evaluate the performance of the proposed method, the AUC values were computed whose averages of* Yeast*,* Human*, and* H. pylori* datasets are 96.29%, 96.47%, and 89.85%, respectively.

The high accuracies show that WSRC-based model combining the SMR-DCT descriptors is feasible and effective for predicting PPIs. In addition, the low standard deviations of these criterion values illustrate that the proposed method is stable and robust. This good performance lies in the fact that the feature extraction method not only depicts the order information of protein sequences but also retains sufficient prior information from BLOSUM62 matrix, which depicts the observed substitutions found in a broad sampling from the aligned segments of polypeptides. In addition, discrete cosine transform performs well in capturing effective information from SMR matrixes. In bioinformatics and evolutionary biology, the substitution matrix like BLOSUM62 describes the rate where one character in a sequence changes to other character states over time. Since the process of the formation of protein interaction network contains innumerable amino acid variations, the substitution rates would help to reveal whether two proteins interact. In fact, protein pairs with higher similarity are more likely to interact and the similarity between protein sequences depends on their divergence time and the substitution rates. The proposed feature extraction method uses this evolutionary information and therefore is able to predict protein-protein interactions.

### 3.3. Comparison with SVM-Based Method

Many machine learning models haven been explored for predicting PPIs and most of them are based on traditional classifiers. To further evaluate the proposed method, we compared it with the state-of-the-art support vector machine (SVM) classifier. Specifically, we used the same feature extraction method and compared the classification performances between SVM and WSRC. We used LIBSVM tool which is available on https://www.csie.ntu.edu.tw/~cjlin/libsvmtools/. A grid search method was used to optimize two corresponding parameters of SVM *c* and *g*. In the experiments of* Yeast* and* Human* dataset, we set *c* = 0.5, *g* = 0.6 and *c* = 0.5, *g* = 0.5, respectively. When exploring on* H. pylori* dataset, we set *c* = 0.08, *g* = 22. The kernel functions were set to be radial basis function.

**Figure 2:**
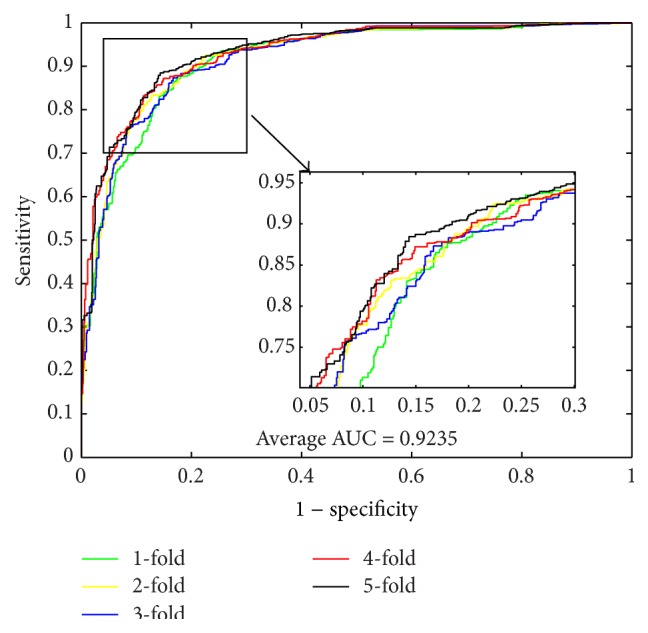
ROC curves performed by SVM-based method on* Yeast* PPIs dataset.

**Figure 3:**
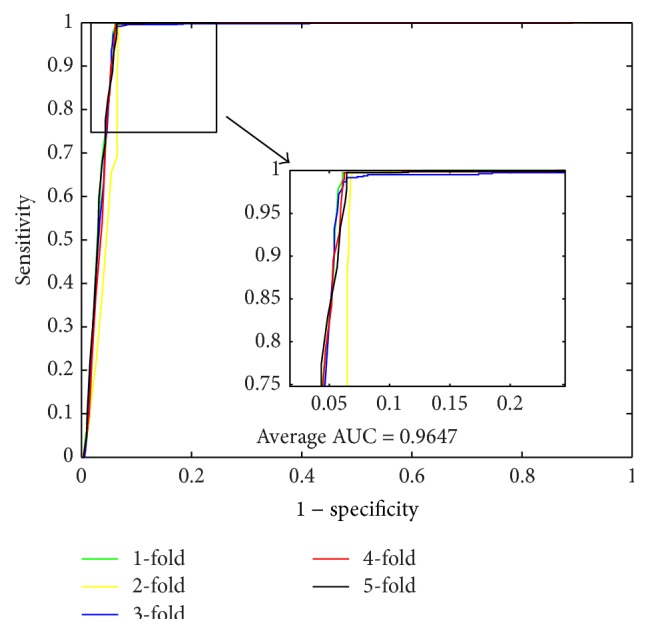
ROC curves performed by proposed method on* Human* PPIs dataset.

**Figure 4:**
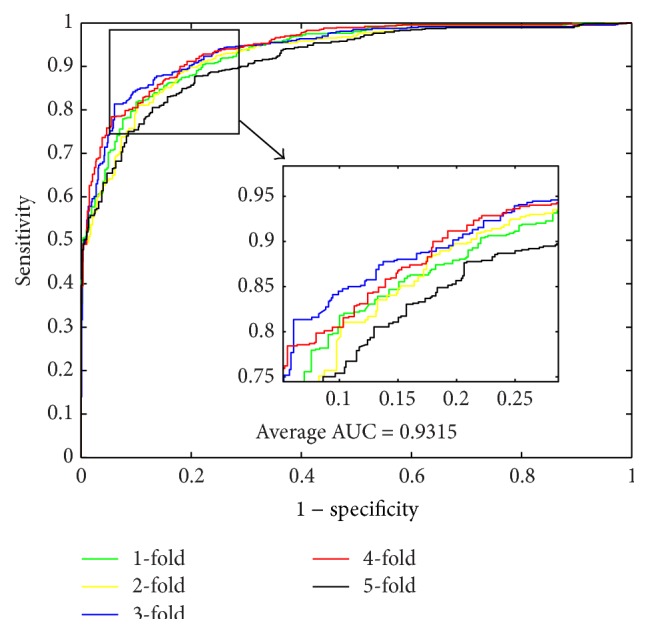
ROC curves performed by SVM-based method on* Human* PPIs dataset.

**Figure 5:**
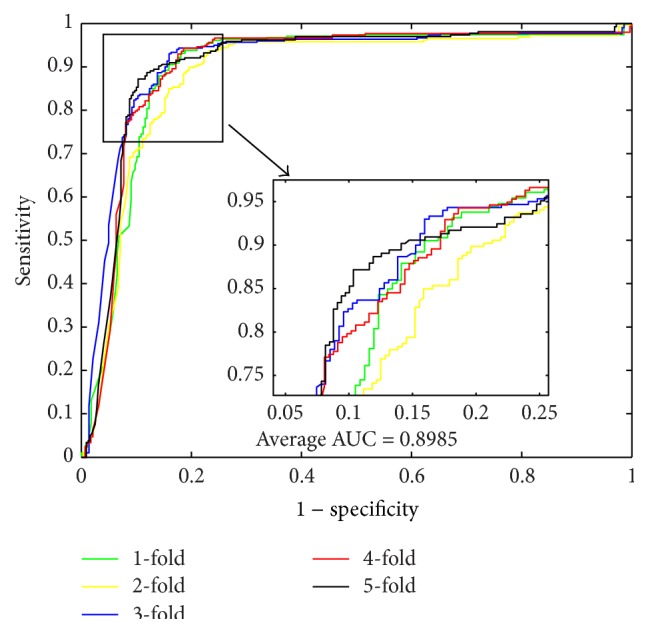
ROC curves performed by proposed method on* H. pylori* PPIs dataset.

**Figure 6:**
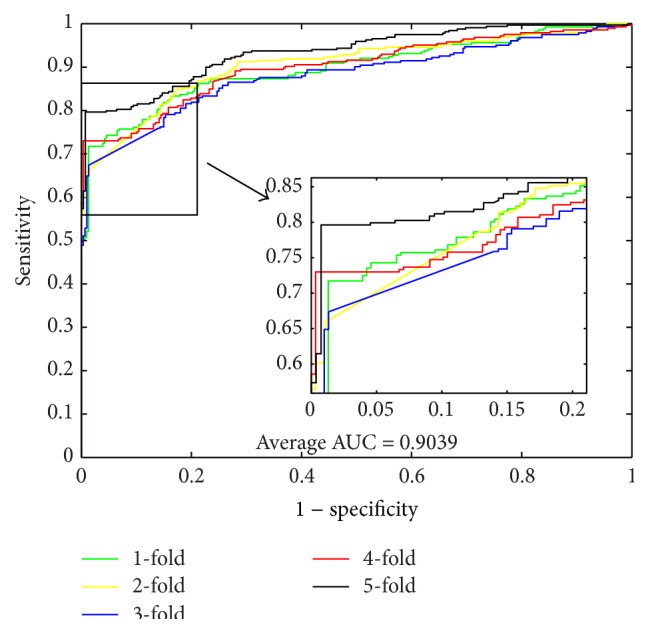
ROC curves performed by SVM-based method on* H. pylori* PPIs dataset.

From Table 4, it can be observed that, when using SVM to predict PPIs of* Yeast* dataset, we obtained good results with the average accuracy, precision, sensitivity, MCC, and AUC of 84.97%, 85.46%, 84.30%, 74.46%, and 92.35%, respectively. When predicting PPIs of* Human* dataset, the SVM-based method yielded good results with the average accuracy, precision, sensitivity, MCC, and AUC of 85.33%, 86.92%, 81.59%, 74.81%, and 93.15%, respectively. When exploring the* H. pylori* dataset, the averages of accuracy, precision, sensitivity, and MCC come to be 80.67%, 83.18%, 79.89%, 67.69%, and 90.39%, respectively. For the three datasets, most of the average criterion values performed by SVM-based method are lower than those by the proposed method. In addition, the higher standard deviations of the criterion values illustrate that the SVM-based model are less stable. The ROC curves performed by SVM classifier on the three datasets are shown in Figures 2, 4, and 6.

Analyzing these results, we can see that sparse representation based classifier is suitable for the classification with protein consequence features. The better performance than SVM lies in the fact that weighted SRC further improves the performance of basic SRC and the easily adjusted parameter of WSRC helps itself giving a full play to its function in our experiments. Therefore, weighted sparse representation based classifier is superior to support vector machine classifier.

### 3.4. Performance on Independent Dataset

As our proposed model yields good performance on the PPIs data of* Yeast*,* Human*, and* H. pylori*, we explored our method on five other independent datasets. It should be noticed that the biological hypothesis of mapping PPIs from one species to another species is that large numbers of physically interacting proteins in one organism have “coevolved” so that their respective orthologs in other organisms interact as well. In these experiments, we used all 11188 samples of* Yeast* dataset as the training set with the optimal parameters (*σ* = 1.5 and *ε* = 0.00005). The same SMR-based feature extraction method was used to transform the protein pairs from the other five datasets into feature vectors as the testing inputs of WSRC. The performance of these five experiments is summarized in Table 5. Four datasets including* E. coli*,* C. elegans*,* H. sapiens*, and* M. musculus* are collected from the DIP database. All of the test samples are positive. When predicting the PPIs on datasets of* E. coli*,* C. elegans*,* H. sapiens*,* H. pylori*, and* M. musculus*, our model yielded accuracies of 66.08%, 81.19%, 82.22%, 82.18%, and 79.87%, respectively. It shows that the metamodel is capable of predicting the PPIs from other species with accuracies of over 66%. When predicting the PPIs of* H. sapiens* and* H. pylori* datasets, we even obtained high accuracies of 82.22% and 82.18%.

Interestingly, these results demonstrate that the information of yeast protein sequences is sufficient for predicting the PPIs of other species. In addition, it implies that the proposed method has strong generalization ability on predicting protein-protein interaction. This model may be applied to exploring the organisms whose PPIs data are not available and provides appropriate experience for further studies.

### 3.5. Comparison with Other Methods

Many computational methods have been proposed for predicting PPIs. Here, we compare the prediction ability of the WSRC prediction model using substitution representation matrix based features with the existing methods on* Yeast* and* H. pylori* datasets.  Table 6 shows the results performed by six other methods and we can see that the accuracies obtained by these methods are between 75.08% and 93.92%. None of these methods gets higher average accuracy than that of the proposed method, which is 96.28%. The same is true for considering precision and sensitivity. Further, the relatively low standard deviations of these criteria values imply the robust performance of the proposed method. From Table 7, we can see the comparison between the proposed method and other previous works on* H. pylori* dataset. The accuracies performed by other methods are between 75.80% and 87.50%. From Table 8, it can be observed that our method yields good results similar to or even better than some other existing methods based on ensemble classifiers.

It is known that the methods which use ensemble classifier usually achieve more accurate and robust performance than the methods using single classifier. However, our proposed model obtains good performance similar to or even better than those obtained by the methods using ensemble classifier, such as Random Forest and ensemble of HKNN, by using the single weighted representation based classifier. Considering these comparisons, it is demonstrated that the WSRC-based model combining the substitution representation matrix based features can improve the prediction accuracy compared with the current state-of-the-art methods. This improvement mainly comes from the choice of classifier and the novel feature extraction method which contains the evolutionary information.

## 4. Conclusions and Discussion

The growing demand for PPIs knowledge is promoting the development of studies on computational methods for predicting PPIs in this postgenomic era. In this paper, we explore a prediction model for PPIs combining weighted sparse representation based classifier and a novel protein representation. In the step of feature extraction, employing discrete cosine transform to extract feature vector from SMR matrix based on BLOSUM62 has been proven effective to represent amino acid sequences. Compared with the earlier methods, the main improvements come from adopting a novel protein feature representation and from using a powerful classifier. Besides, results show that it is feasible to use weighted sparse representation based classifier to deal with protein features. Further, experiments on other independent protein datasets imply the powerful generalization ability of the proposed method. Hence, we consider that our proposed method is feasible, superior, and robust.

## Figures and Tables

**Table 1:** 5-fold cross-validation results obtained by using proposed method on *Yeast* dataset.

Testing set	Accu. (%)	Prec. (%)	Sen. (%)	MCC (%)	AUC (%)
1	96.74	100.00	93.60	93.68	97.07
2	95.89	100.00	91.93	92.10	96.04
3	96.92	100.00	93.75	94.00	96.83
4	95.75	100.00	91.50	91.84	95.49
5	96.12	99.60	92.39	92.50	96.03
Average	**96.28 ± 0.52**	**99.92 ± 0.18**	**92.64 ± 1.00**	**92.82 ± 0.97**	**96.29 ± 0.65**

**Table 2 tab2:** 5-fold cross-validation results obtained by using proposed method on *Human *dataset.

Testing set	Accu. (%)	Prec. (%)	Sen. (%)	MCC (%)	AUC (%)
1	96.20	99.73	92.53	92.66	96.85
2	96.32	99.72	92.48	92.85	95.52
3	96.32	99.06	93.32	92.89	96.59
4	96.45	99.72	92.73	93.08	96.60
5	96.20	99.72	92.12	92.61	96.78
Average	**96.30 ± 0.10**	**99.59 ± 0.29**	**92.63 ± 0.44**	**92.82 ± 0.19**	**96.47 ± 0.54**

**Table 3:** 5-fold cross-validation results obtained by using proposed method on *H. pylori* dataset.

Testing set	Accu. (%)	Prec. (%)	Sen. (%)	MCC (%)	AUC (%)
1	86.08	84.95	85.87	75.99	89.42
2	84.71	84.62	85.47	74.08	88.08
3	88.83	89.17	87.59	80.13	91.11
4	87.29	87.02	87.02	77.79	90.15
5	86.82	89.29	86.21	76.97	90.48
Average	**86.74 ± 1.52**	**87.01 ± 2.23**	**86.43 ± 0.86**	**76.99 ± 2.23**	**89.85 ± 1.16**

**Table 4:** Comparison with support vector machine on three datasets.

Dataset	Classifier	Accu. (%)	Prec. (%)	Sen. (%)	MCC (%)	AUC (%)
*Yeast*	WSRC	**96.28 ± 0.52**	**99.92 ± 0.18**	**92.64 ± 1.00**	**92.82 ± 0.97**	**96.29 ± 0.65**
	SVM	84.97 ± 0.93	85.46 ± 1.21	84.30 ± 0.83	74.46 ± 1.29	92.35 ± 0.72

*Human*	WSRC	**96.30 ± 0.10**	**99.59 ± 0.29**	**92.63 ± 0.44**	**92.82 ± 0.19**	**96.47 ± 0.54**
	SVM	85.33 ± 1.29	86.92 ± 1.92	81.59 ± 2.40	74.81 ± 1.89	93.15 ± 1.11

*H. pylori*	WSRC	**86.74 ± 1.52**	**87.01 ± 2.23**	**86.43 ± 0.86**	**76.99 ± 2.23**	**89.85 ± 1.16**
	SVM	80.67 ± 1.95	83.18 ± 9.85	79.89 ± 11.83	67.69 ± 3.33	90.39 ± 1.91

**Table 5:** Prediction results on five species based on our model.

Species	Test pairs	Accuracy
*E. coli*	6954	66.08%
*C. elegans*	4013	81.19%
*H. sapiens*	**1412**	**82.22%**
*H. pylori*	**1420**	**82.18%**
*M. musculus*	313	79.87%

**Table 6:** Performance comparison of different methods on the *Yeast* dataset.

Model	Test set	Accu. (%)	Prec. (%)	Sen. (%)	MCC (%)
Guos' work [25]	ACC	89.33 ± 2.67	88.87 ± 6.16	89.93 ± 3.68	N/A
	AC	87.36 ± 1.38	87.82 ± 4.33	87.30 ± 4.68	N/A

Zhous' work [26]	SVM + LD	88.56 ± 0.33	89.50 ± 0.60	87.37 ± 0.22	77.15 ± 0.68

Yangs' work [27]	Cod1	75.08 ± 1.13	74.75 ± 1.23	75.81 ± 1.20	N/A
	Cod2	80.04 ± 1.06	82.17 ± 1.35	76.77 ± 0.69	N/A
	Cod3	80.41 ± 0.47	81.86 ± 0.99	78.14 ± 0.90	N/A
	Cod4	86.15 ± 1.17	90.24 ± 1.34	81.03 ± 1.74	N/A

Wongs' work [28]	RF + PR-LPQ	93.92 ± 0.36	96.45 ± 0.45	91.10 ± 0.31	88.56 ± 0.63

Yous' work [29]	PCA-EELM	87.00 ± 0.29	87.59 ± 0.32	86.15 ± 0.43	77.36 ± 0.44

Proposed method	**WSRC**	**96.28 ± 0.52**	**99.92 ± 0.18**	**92.64 ± 1.00**	**92.82 ± 0.97**

**Table 7:** Performance comparison of different methods on the *H. pylori* dataset.

Model	Accu. (%)	Prec. (%)	Sen. (%)	MCC (%)
Phylogenetic bootstrap [30]	75.80	80.20	69.80	N/A
HKNN [31]	84.00	84.00	86.00	N/A
Signature products [32]	83.40	85.70	79.90	N/A
Ensemble of HKNN [33]	86.60	85.00	86.70	N/A
Boosting [34]	79.52	81.69	80.37	70.64
Ensemble ELM [29]	87.50	86.15	88.95	78.13
Proposed method	**86.74**	**87.01**	**86.43**	**76.99**

**Table 8:** Performance comparison of different methods on the *Human* dataset.

Model	Accu. (%)	Prec. (%)	Sen. (%)	MCC (%)
LDA + RF [35]	96.4	N/A	94.2	92.8
LDA + RoF [35]	95.7	N/A	97.6	91.8
LDA + SVM [35]	90.7	N/A	89.7	81.3
AC + RF [35]	95.5	N/A	94.0	91.4
AC + RoF [35]	95.1	N/A	93.3	91.0
AC + SVM [35]	89.3	N/A	94.0	79.2
Proposed method	**96.30**	**99.59**	**92.63**	**92.82**
